# Atopic Features and Inflammatory Markers Across Cassano-Graded Adenoid Hypertrophy

**DOI:** 10.3390/children13030374

**Published:** 2026-03-06

**Authors:** Fatih Kaplan, Bilge Kurnaz Kaplan, Abdulgani Gülyüz

**Affiliations:** 1 Department of Pediatric Allergy and Immunology, Malatya Training and Research Hospital, 44000 Malatya, Türkiye; drfthkpln@gmail.com; 2 Department of Otorhinolaryngology, Malatya Training and Research Hospital, 44000 Malatya, Türkiye; drblgkpln@gmail.com; 3 Department of Pediatrics, Faculty of Medicine, Malatya Turgut Özal University, 44210 Malatya, Türkiye

**Keywords:** adenoid hypertrophy, Cassano classification, eosinophilia, total IgE, sensitization, allergic rhinitis, pediatric

## Abstract

**Highlights:**

**What are the main findings?**

• In children with adenoid hypertrophy, disease severity was independently associated with eosinophilia rather than with IgE-mediated sensitization.
• Family history of atopy and elevated total IgE were the strongest factors associated with clinical atopy.

**What is the implication of the main finding?**

• Inflammatory burden may play a more important role than classical allergic sensitization in determining adenoid hypertrophy severity.
• Evaluation of eosinophilia may provide additional clinical information in the routine assessment of children with adenoid hypertrophy.

**Abstract:**

Background: Evidence linking adenoid hypertrophy (AH) and atopy is conflicting. We examined whether Cassano-graded AH severity is more closely associated with inflammatory markers than with IgE-mediated sensitization. Methods: We retrospectively included children aged 3–12 years diagnosed with AH between December 2022 and December 2025. AH was graded according to the Cassano classification and dichotomized as advanced AH (Stage III–IV). Atopic features were evaluated separately as clinical atopy, IgE-mediated sensitization, elevated total IgE, and eosinophilia. Multivariable logistic regression analyses were performed to assess factors associated with clinical atopy, sensitization, and advanced AH. Results: Among 426 children, clinical atopy was present in 28.2%, sensitization in 23.0%, elevated total IgE in 16.4%, and eosinophilia in 27.7%; 39.2% had advanced AH. In multivariable analysis, clinical atopy was independently associated with family history of atopy (aOR 13.9; 95% CI 7.9–24.4), elevated total IgE (aOR 3.86; 95% CI 2.10–7.08), and passive smoking exposure (aOR 1.73; 95% CI 1.07–2.79). Sensitization was independently associated only with family history of atopy (aOR 4.99; 95% CI 1.99–12.53). Advanced AH was independently associated only with eosinophilia (aOR 2.07; 95% CI 1.30–3.29). Conclusions: AH severity was associated with eosinophilia rather than classical IgE-mediated sensitization. Assessment of eosinophilia may aid routine severity evaluation in children with AH.

## 1. Introduction

Adenoid tissue is a physiological lymphoid structure located in the nasopharynx during childhood and plays a crucial role in shaping immune responses, particularly in early life [1]. Hypertrophy of this tissue is considered one of the most common causes of upper airway obstruction and is associated with clinical manifestations such as nasal breathing difficulty, snoring, sleep disturbances, and recurrent upper respiratory tract infections [2]. In addition to infectious factors, immunological and inflammatory mechanisms are known to contribute to the development of adenoid hypertrophy [3,4].

Atopy represents a heterogeneous immunological spectrum encompassing clinical conditions such as allergic rhinitis, asthma, and atopic dermatitis, as well as biological markers including aeroallergen sensitization, elevated total immunoglobulin E (IgE) levels, and eosinophilia [5]. In childhood, atopic features may be characterized by chronic inflammation of the upper and lower airways, which may contribute to enlargement of nasopharyngeal lymphoid tissue and persistence of symptoms [6,7]. In this context, adenoid hypertrophy may reflect not only mechanical obstruction but also an anatomical manifestation of the underlying immunological burden [6].

Previous studies investigating the relationship between adenoid hypertrophy and atopic features have reported conflicting results. Some studies have demonstrated that adenoid hypertrophy is more frequent and more severe in children diagnosed with allergic rhinitis or asthma, with this association generally interpreted on the basis of clinical diagnoses or total IgE levels [8]. In contrast, other studies based on aeroallergen sensitization or serum IgE levels have failed to demonstrate a significant association with adenoid hypertrophy [9]. Furthermore, studies that evaluated adenoid hypertrophy merely as present or absent, without grading disease severity, have yielded inconsistent findings [10]. These discrepancies may be related to differences in the definition of atopy, the lack of integrated evaluation of biological markers, and the absence of graded analysis of adenoid hypertrophy.

The Cassano classification, which is widely used to assess the degree of adenoid hypertrophy, enables functional evaluation of the extent of nasopharyngeal obstruction caused by adenoid tissue [11]. However, studies examining the degree of adenoid hypertrophy together with the underlying atopic burden using a graded approach remain limited. In particular, evaluating biological markers such as aeroallergen sensitization, total IgE levels, and eosinophilia alongside the severity of adenoid hypertrophy may contribute to a better understanding of the pathophysiological basis of this relationship [8,11].

This study focused on routinely available peripheral inflammatory markers that are easily obtainable in clinical practice and therefore have strong real-world relevance. Rather than comparing children with adenoid hypertrophy to healthy controls, it aimed to explore associations between disease severity and atopic features within an AH population.

The aim of this study was to evaluate the distribution of atopic features in children diagnosed with adenoid hypertrophy, to examine the relationship between the degree of adenoid hypertrophy and atopic burden, and to identify clinical and laboratory factors associated with the presence of atopy. Specifically, we investigated whether the severity of adenoid hypertrophy is associated with inflammatory biological markers rather than with classical IgE-mediated sensitization indicators. Based on retrospective data from the past three years, this study aims to present a comprehensive approach integrating both clinical and laboratory findings.

## 2. Materials and Methods

### 2.1. Study Design and Patient Selection

This study was conducted through a retrospective review of the medical records of patients who presented to the otorhinolaryngology (ENT) and pediatric allergy outpatient clinics of a tertiary care center. Pediatric patients diagnosed with adenoid hypertrophy between December 2022 and December 2025 were included.

Patients presenting to the ENT outpatient clinic with complaints of nasal obstruction, snoring, mouth breathing, and/or recurrent upper respiratory tract infections were evaluated for adenoid hypertrophy following clinical assessment and flexible nasopharyngeal endoscopy. Patients diagnosed with adenoid hypertrophy were referred to the pediatric allergy clinic for evaluation of accompanying atopic features. Conversely, patients presenting to the pediatric allergy clinic with suspected allergic rhinitis, asthma, or atopic dermatitis and with findings suggestive of adenoid hypertrophy on history or physical examination were referred to the ENT clinic for adenoid assessment. Patients in whom the diagnosis of adenoid hypertrophy was confirmed through this bidirectional clinical evaluation process were included in the study.

### 2.2. Inclusion and Exclusion Criteria

**Figure 1:**
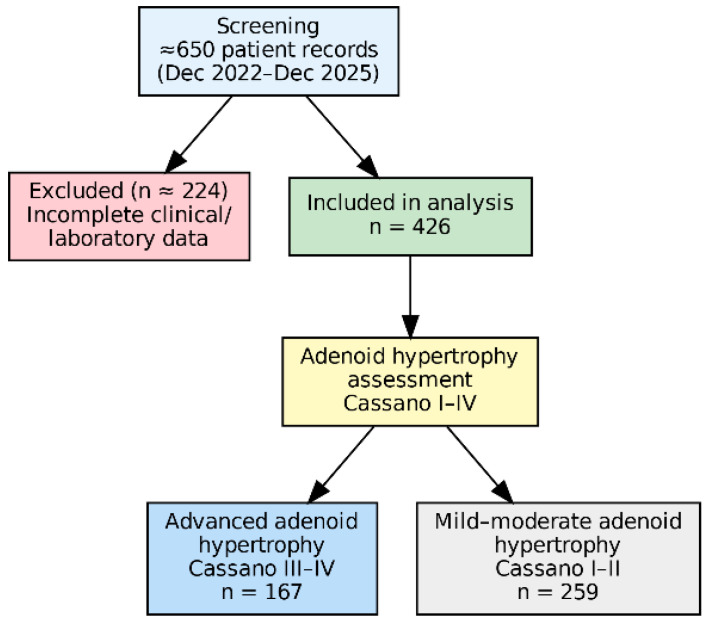
Flow diagram of patient selection. Of 650 pediatric patients assessed for eligibility, 426 with complete clinical and laboratory data were included in the final analysis. Patients with missing data were excluded.

Patients aged 3–12 years with a diagnosis of adenoid hypertrophy were included. Patients with a history of adenoidectomy, craniofacial anomalies, systemic inflammatory diseases, or incomplete clinical or laboratory data were excluded. The number of patients excluded due to missing data and the reasons for exclusion are presented in the patient flow diagram (Figure 1).

### 2.3. Assessment of Adenoid Hypertrophy

The degree of adenoid hypertrophy was determined using the Cassano classification based on findings from flexible nasopharyngeal endoscopy performed in the ENT outpatient clinic [11]. According to this classification, the extent of choanal obstruction caused by adenoid tissue was graded from Stage I to Stage IV. Evaluations were performed by experienced ENT specialists as part of routine clinical practice, and no additional procedures were undertaken for the purposes of the study.

In statistical analyses, the degree of adenoid hypertrophy was evaluated both as an ordinal categorical variable and in dichotomized form as Cassano Stage III–IV versus Stage I–II to represent clinically significant advanced hypertrophy.

### 2.4. Assessment of Atopic Features

#### 2.4.1. Clinical Atopy Definition

Clinical atopy was defined as the presence of physician-diagnosed allergic rhinitis, asthma, atopic dermatitis, and/or food allergy, representing clinically manifest allergic disease. Allergic rhinitis was diagnosed according to the Allergic Rhinitis and its Impact on Asthma (ARIA) guidelines [5], asthma according to the Global Initiative for Asthma (GINA) recommendations [12], and atopic dermatitis according to the Hanifin–Rajka criteria, with disease severity assessed using the SCORAD index [13,14].

#### 2.4.2. IgE-Mediated Sensitization Definition

IgE-mediated sensitization was defined as the presence of aeroallergen sensitization demonstrated by a positive skin prick test and/or elevated serum-specific IgE levels [5,6]. Sensitization status was evaluated independently of clinical symptoms.

#### 2.4.3. Eosinophilia Definition (Inflammatory Marker)

Eosinophilia was defined as an absolute eosinophil count above age-specific reference ranges and was evaluated as a dichotomous variable (present/absent). Eosinophilia was considered a biological marker of type-2 inflammatory activity rather than a diagnostic criterion for atopy [6].

These components were analyzed as separate variables in all statistical models to avoid conceptual overlap between clinical atopy, sensitization, and inflammatory markers.

Given limited availability of allergen-specific perennial/seasonal categorization, sensitization pattern was approximated using ARIA duration of allergic rhinitis (intermittent vs. persistent) for stratified analyses.

### 2.5. Clinical Data and Treatment Approach

Medical treatment data were obtained from patient records. Treatment approaches were categorized as follow-up only, medical therapy, surgery after failure of medical therapy, and primary surgery. Medical therapy included the use of intranasal corticosteroids, antihistamines, and/or montelukast, either alone or in combination. Passive smoke exposure and family history of atopy were obtained from medical records.

### 2.6. Statistical Analysis

Statistical analyses were performed using IBM SPSS Statistics for Windows, Version 26 (IBM Corp., Armonk, NY, USA). Continuous variables were presented as median and interquartile range (IQR), and categorical variables as number and percentage (%). Group comparisons were performed using the Mann–Whitney U test, chi-square test, or Fisher’s exact test, as appropriate. Chi-square test for trend was applied for ordinal categorical variables.

Multivariable logistic regression models were constructed to evaluate the presence of atopy and advanced adenoid hypertrophy (Cassano Stage III–IV). Results were reported as adjusted odds ratios (aORs), 95% confidence intervals, and *p*-values. Model fit was assessed using the Hosmer–Lemeshow test. A *p*-value < 0.05 was considered statistically significant. The models were constructed to assess associations and were not intended for the development of a predictive risk score.

Because of the retrospective design, treatment approach may represent a consequence of disease severity and could introduce confounding by indication; therefore, it was not included in the regression models.

Additional multivariable logistic regression analyses were performed stratified by predefined age groups (3–5, 6–8, and 9–12 years) to explore potential age-related effect modification.

### 2.7. Ethical Approval

The study was approved by the institutional ethics committee. Due to the retrospective design, the requirement for informed consent was waived by the ethics committee.

## 3. Results

### 3.1. Patient Characteristics

A total of 426 children followed with a diagnosis of adenoid hypertrophy were included in this study. The age distribution of the study population encompassed different stages of childhood, and demographic as well as clinical characteristics were evaluated in detail. Male and female children were represented in similar proportions. Passive smoke exposure was present in a subset of patients and was considered in further analyses due to its potential association with both atopy and adenoid hypertrophy (Table 1).

Across age strata, clinical atopy and sensitization were more frequent in the 9–12-year group, while eosinophilia also showed an increasing tendency with age (Table S1).

### 3.2. Degree of Adenoid Hypertrophy

Adenoid hypertrophy was graded according to the Cassano classification [11]. While a portion of the patients were classified as Cassano Stage I–II, 167 patients (39.2%) were classified as Cassano Stage III–IV and were considered to have advanced adenoid hypertrophy. This distribution enabled analyses focusing not only on the presence but also on the severity of adenoid hypertrophy, and advanced hypertrophy was used as the primary dependent variable in subsequent statistical evaluations (Table 1).

### 3.3. Distribution of Atopic Features

Clinical atopy was present in 120 of 426 patients (28.2%). Aeroallergen sensitization was detected in 23.0% of patients. Elevated total IgE levels were observed in 16.4%, and eosinophilia in 27.7% of patients.

**Figure 2:**
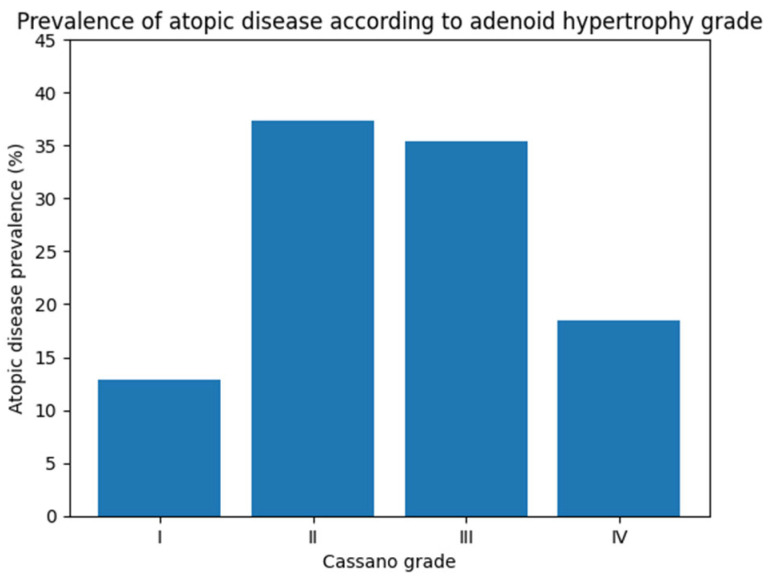
Distribution of clinical atopy prevalence across adenoid hypertrophy grades according to the Cassano classification. Bars represent the percentage of patients with atopy within each Cassano grade. Clinical atopy was defined as the presence of physician-diagnosed allergic rhinitis, asthma, atopic dermatitis, and/or food allergy.

The distribution of atopic features within the study group was evaluated in detail (Table 2). Aeroallergen sensitization was detected in 23.0% of patients. Total immunoglobulin E levels, assessed according to age-adjusted reference ranges, were elevated in 16.4% of cases. Eosinophilia, defined based on the absolute eosinophil count, was present in 27.7% of patients. The prevalence of atopy across Cassano stages is illustrated in Figure 2.

Atopic dermatitis was identified in 6.8% of patients, with the majority classified as mild to moderate in severity. Allergic rhinitis was observed in 22.1% of patients; among those diagnosed with allergic rhinitis, 51.1% had intermittent and 48.9% had persistent disease. Asthma was present in 9.2% of patients. Food allergy was detected in 4.0% and, due to its low prevalence, was considered a descriptive characteristic (Table 2).

When atopic characteristics were evaluated across Cassano stages, a significant linear trend was observed for eosinophilia and aeroallergen sensitization (*p* for trend < 0.001 for both; Table 3). In contrast, the trend for elevated total IgE across stages did not reach statistical significance (*p* for trend = 0.062). Clinical atopy prevalence did not increase monotonically with stage; it rose from Stage I to Stages II–III and decreased in Stage IV (*p* for trend < 0.001; Table 3). Despite the significant trend test, the pattern was non-monotonic.

### 3.4. Factors Associated with Atopy

Two separate multivariable logistic regression models were constructed to identify factors associated with clinical atopy and IgE-mediated sensitization (Table 4A,B).

In the multivariable model evaluating clinical atopy (Table 4A), family history of atopy (aOR 13.9; 95% CI 7.9–24.4), elevated total IgE (aOR 3.86; 95% CI 2.10–7.08), and passive smoking exposure (aOR 1.73; 95% CI 1.07–2.79) were independently associated with clinical atopy. Age, sex, eosinophilia, and advanced adenoid hypertrophy (Cassano Stages III–IV) were not independently associated.

In the model evaluating IgE-mediated sensitization, only family history of atopy remained independently associated (aOR 4.99; 95% CI 1.99–12.53; Table 4B). Age, sex, passive smoking exposure, elevated total IgE, eosinophilia, and advanced adenoid hypertrophy were not independently associated with sensitization.

Regarding treatment approaches, 56.1% of patients received medical therapy, 15.7% were referred for surgery due to failure of medical treatment, 6.3% underwent primary surgery, and 21.8% were managed with follow-up alone.

### 3.5. Factors Associated with Advanced Adenoid Hypertrophy

A separate multivariable logistic regression model was constructed to evaluate factors associated with advanced adenoid hypertrophy (Cassano Stage III–IV) (Table 5). This model included age, sex, passive smoke exposure, elevated total immunoglobulin E, aeroallergen sensitization, eosinophilia, and family history of atopy. In multivariable analysis, eosinophilia was the only variable independently associated with advanced adenoid hypertrophy (aOR 2.07; 95% CI 1.30–3.29; *p* = 0.002). Other variables, including elevated total immunoglobulin E and aeroallergen sensitization, were not independently associated with advanced adenoid hypertrophy. This exploratory comparison was restricted to children with allergic rhinitis for whom ARIA duration data (intermittent vs. persistent) were available. Persistent (perennial-pattern) rhinitis was more common in advanced AH (Cassano 3–4: 23/39, 59.0%) compared with mild–moderate AH (Cassano 1–2: 21/53, 39.6%), although this did not reach statistical significance in exploratory subgroup analyses (Fisher’s exact *p* = 0.091; Supplementary Table S1).

In age-stratified analyses, eosinophilia remained significantly associated with advanced adenoid hypertrophy in the 6–8-year age group (aOR 3.54; 95% CI 1.60–7.83; *p* = 0.002; Table S2), whereas no independent associations were observed in the younger (3–5 years) or older (9–12 years) groups.

Available records suggested that perennial-pattern sensitization (including house dust mite) was uncommon; however, sensitization subtype data were incomplete and should be interpreted cautiously.

## 4. Discussion

In this study, the severity of adenoid hypertrophy in children was more closely associated with inflammatory burden—particularly eosinophilia—than with classical IgE-mediated sensitization. This finding may help to clarify the conflicting results in the literature regarding the relationship between adenoid hypertrophy and atopy [15,16].

In the existing literature, the relationship between adenoid hypertrophy and atopy has largely been examined using a dichotomous (present/absent) approach. While some studies have reported associations between allergic rhinitis, elevated total IgE levels, or aeroallergen sensitization and adenoid hypertrophy, others have failed to demonstrate such relationships [17,18,19]. Potential explanations for these inconsistencies include the absence of graded assessment of adenoid hypertrophy, failure to evaluate biological markers within a unified analytical framework, and inadequate control of potential confounders. In our study, adenoid hypertrophy was graded according to the Cassano classification, and both clinical phenotypes and laboratory markers were analyzed simultaneously in multivariable models. The classification of Cassano Stage III–IV as advanced adenoid hypertrophy was based on clinical practice and prior studies; however, sensitivity analyses using alternative classifications may be informative in future research [20]. In this regard, our study seeks to address a methodological gap in the existing literature.

The prevalence of atopy did not demonstrate a monotonic increase across Cassano stages; instead, rates rose from Stage I to Stages II–III and declined in Stage IV (Table 3). This non-linear pattern should be interpreted with caution, as the relatively smaller number of Stage IV cases and the retrospective, clinic-based sampling design may have influenced the observed distribution rather than reflecting a true biological gradient. Notably, eosinophilia was the only variable independently associated with advanced adenoid hypertrophy in the multivariable analysis, suggesting that inflammatory burden—rather than clinically defined atopy—may be more closely related to disease severity [21,22].

In the model focusing on clinical atopy, elevated total IgE levels were independently associated with atopy, which is an expected finding. Treatment patterns likely reflect symptom burden and clinical recognition rather than a causal effect; therefore, treatment was described descriptively and was not included in the regression models due to confounding by indication [18,23]. In contrast, the absence of an independent association between eosinophilia and atopy suggests that eosinophilia may reflect inflammatory burden and tissue-level responses rather than clinically defined atopy. Adenoid tissue has been shown to harbor concurrent viral infections and bacterial colonization, which may sustain chronic local inflammatory responses independent of systemic IgE-mediated sensitization [24]. The low prevalence of perennial sensitization, particularly house dust mite allergy, may reflect regional environmental characteristics and limits definitive conclusions regarding the role of chronic allergen exposure in adenoid hypertrophy.

The association between eosinophilia and adenoid hypertrophy severity appeared to be most pronounced in the mid-childhood group (6–8 years), suggesting a potential age-related window during which type-2 inflammatory mechanisms may play a greater role in lymphoid tissue enlargement. However, the absence of consistent findings across all age strata indicates that this relationship should be interpreted with caution.

In the model evaluating factors associated with advanced adenoid hypertrophy, eosinophilia emerged as the only independently significant variable, representing one of the most notable findings of the study. In this analysis, total IgE levels and aeroallergen sensitization lost statistical significance, suggesting that the severity of adenoid hypertrophy may be more closely related to local or systemic inflammatory cellular responses than to IgE-mediated systemic sensitization [21,22]. This finding further indicates that crude associations observed in trend analyses may attenuate when biological markers and potential confounders are evaluated simultaneously in multivariable models. The absence of an association between eosinophilia and the “presence of atopy” model further supports the notion that eosinophilia may serve as a marker of inflammatory burden rather than of clinically defined atopy.

Eosinophils are known to participate in type-2 inflammatory processes in the upper airway; however, the present findings reflect systemic associations rather than direct evidence of tissue-level mechanisms.

Secondary analyses of comorbid allergic conditions—including rhinitis severity, food allergy, and asthma—did not demonstrate consistent independent associations with AH severity and should therefore be interpreted as exploratory, particularly given the limited sample sizes in subgroup analyses. Food allergy was infrequent in our cohort and was therefore evaluated descriptively. Because assessment relied primarily on clinical records and IgE-based testing, non-IgE-mediated food allergy may have been underrecognized; therefore, its potential contribution to adenoid hypertrophy severity cannot be excluded.

In conclusion, this study suggests that the severity of adenoid hypertrophy may be associated with inflammatory markers independently of classical IgE-mediated sensitization. These findings may provide a basis for more comprehensive and biologically grounded approaches to assessing disease severity in children with adenoid hypertrophy.

## 5. Conclusions

Atopic features are common in children with adenoid hypertrophy; however, eosinophilia—rather than classical IgE-mediated sensitization—was independently associated with advanced disease. These findings suggest that inflammatory burden may play a more prominent role in determining disease severity than IgE-based markers alone. Assessment of eosinophilia may therefore provide clinically relevant information in the evaluation of children with adenoid hypertrophy.

## 6. Limitations and Strengths

This study has several strengths. It includes a relatively large cohort of children with adenoid hypertrophy and applies a graded endoscopic classification based on the Cassano system, allowing evaluation of disease severity rather than relying on a simple presence–absence approach. Clinical atopic phenotypes and biological markers were assessed simultaneously within multivariable models, enabling adjustment for potential confounders. This integrated approach provides a more comprehensive characterization of factors associated with advanced adenoid hypertrophy in routine clinical practice.

This study also has several limitations. Its retrospective, single-center design may limit generalizability. Because only children with adenoid hypertrophy were included and no healthy control group was available, the findings should be interpreted as associations within an AH population rather than as determinants of AH development. The absence of histopathological evaluation and local inflammatory markers, including cytokine profiles, precluded direct assessment of tissue-level immune mechanisms. Selection bias may have occurred due to bidirectional referral between otorhinolaryngology and pediatric allergy clinics. In addition, allergen-specific categorization of sensitization was incomplete, and seasonal or perennial patterns could only be approximated using clinical indicators. Therefore, causal inferences cannot be drawn, and prospective controlled studies incorporating local immunological markers are required to confirm these findings. Subgroup analyses may have been underpowered because of smaller sample sizes within age strata. The relatively small number of sensitized patients may also have limited statistical power in this model.

In addition, food allergy assessment did not include standardized evaluation of non–IgE-mediated forms, which are relatively common in early childhood and may occur without detectable IgE sensitization. Therefore, the contribution of non-IgE-mediated food allergy to adenoid hypertrophy severity could not be assessed.

## 7. Clinical Implications

In children diagnosed with adenoid hypertrophy, particularly those with advanced hypertrophy, comprehensive evaluation for concomitant atopic diseases and immunological markers may be clinically beneficial. Integration of biological indicators, such as total IgE levels and eosinophilia, into clinical decision-making processes may contribute to more individualized follow-up and treatment strategies.

## 8. Future Directions

Prospective, multicenter studies will help to more clearly define the relationship between adenoid hypertrophy and atopic diseases. In addition, evaluation of local adenoid tissue inflammation, local IgE production, and cytokine profiles may help elucidate the underlying pathophysiological mechanisms. Subgroup analyses according to age groups may further clarify developmental differences in atopic and inflammatory responses.

## Figures and Tables

**Table 1:** Baseline characteristics of children with adenoid hypertrophy according to atopy status.

Variable	Total (*n* = 426)	With Atopy (*n* = 120)	Without Atopy (*n* = 306)	*p*
Age (years), median [IQR]	6 (5–8)	7 (5–9)	6 (5–8)	0.078
Sex, n (%)				0.401
Male	232 (54.5)	69 (57.5)	163 (53.3)	
Female	194 (45.5)	51 (42.5)	143 (46.7)	
Exposure to passive smoking, n (%)	103 (24.2)	39 (32.5)	64 (20.9)	0.012
Family history of atopy, n (%)	101 (23.7)	72 (60.0)	29 (9.5)	<0.001
Adenoid grade (Cassano), n (%)				<0.001
Stage I	101 (23.7)	13 (10.8)	88 (28.9)	
Stage II	158 (37.1)	57 (47.5)	101 (33.0)	
Stage III	113 (26.5)	40 (33.3)	73 (23.9)	
Stage IV	54 (12.7)	10 (8.3)	44 (14.4)	
Treatment approach, n (%)				
Follow-up	93 (21.8)			
Medical treatment	239 (56.1)			
Surgery after medical treatment	67 (15.7)			
Primary surgery	27 (6.3)			

Data are presented as median [IQR] or number (percentage). Continuous variables were compared using the Mann–Whitney U test and categorical variables using the chi-square or Fisher’s exact test, as appropriate. Clinical atopy was defined as physician-diagnosed allergic rhinitis, asthma, atopic dermatitis, and/or food allergy. Cassano stages are based on the Cassano classification [11]. Treatment approach is presented descriptively for the entire cohort and was not included in multivariable analyses due to potential confounding by indication. A *p*-value < 0.05 was considered statistically significant.

**Table 2:** Distribution of atopic characteristics among children with adenoid hypertrophy.

Atopic Characteristic	Total (*n* = 426)
Aeroallergen sensitization, n (%)	
Yes	98 (23.0)
No	328 (77.0)
Total IgE level (age-adjusted), n (%)	
Elevated	70 (16.4)
Normal	356 (83.6)
Eosinophilia (AEC ↑), n (%)	
Yes	118 (27.7)
No	308 (72.3)
Atopic dermatitis, n (%)	
Absent	397 (93.2)
Mild (SCORAD 1)	10 (2.3)
Moderate (SCORAD 2)	13 (3.1)
Severe (SCORAD 3)	6 (1.4)
Allergic rhinitis, n (%)	
Absent	332 (77.9)
Present	94 (22.1)
Intermittent	48 (51.1)
Persistent	46 (48.9)
Mild	42 (44.7)
Moderate/Severe	52 (55.3)
Asthma, n (%)	
Yes	39 (9.2)
No	387 (90.8)
Food allergy, n (%)	
Yes	17 (4.0)
No	409 (96.0)

Data are presented as number (percentage). Total IgE levels were classified according to age-adjusted reference ranges. Eosinophilia was defined based on the absolute eosinophil count. Atopic dermatitis severity was assessed using the SCORAD index. Allergic rhinitis severity was classified as mild or moderate–severe according to ARIA criteria; percentages for severity and duration were calculated only among patients with allergic rhinitis (n = 94).

**Table 3:** Association between Cassano grade and atopic characteristics in children with adenoid hypertrophy.

Cassano Grade	Atopy Present (%)	Elevated IgE (%)	Eosinophilia (%)	Aeroallergen Sensitization (%)	SCORAD ≥ 2 (%)	*n*
I	12.9	9.9	15.8	6.9	2.0	101
II	36.1	18.4	25.3	31.0	5.1	158
III	35.4	22.1	41.6	30.1	7.1	113
IV	18.5	11.1	27.8	14.8	1.9	54
*p* for trend	<0.001	0.062	<0.001	<0.001	0.118	

Percentages represent proportions within each Cassano stage. *p* values are from the chi-square test for trend (linear-by-linear association). Clinical atopy was defined as physician-diagnosed allergic rhinitis, asthma, atopic dermatitis, and/or food allergy. Aeroallergen sensitization was defined as a positive skin prick test and/or allergen-specific IgE positivity. SCORAD ≥ 2 indicates moderate-to-severe atopic dermatitis.

**Table 4:** (**A**) Multivariable logistic regression analysis of factors associated with clinical atopy in children with adenoid hypertrophy. (**B**) Multivariable logistic regression analysis of factors associated with IgE-mediated sensitization in children with adenoid hypertrophy.

(**A**)			
**Variable**	**aOR**	**95% CI**	***p***
Age (years)	1.09	0.97–1.23	0.14
Male sex	1.21	0.78–1.87	0.39
Passive smoking	1.73	1.07–2.79	0.025
Family history of atopy	13.9	7.9–24.4	<0.001
Cassano Stage III–IV	0.82	0.52–1.30	0.40
High total IgE	3.86	2.10–7.08	<0.001
Eosinophilia	1.04	0.65–1.65	0.88
(**B**)			
**Independent Variable**	**aOR**	**95% CI**	***p***
Age (years)	1.09	0.91–1.31	0.342
Male sex	0.88	0.37–2.10	0.781
Passive smoking	1.45	0.54–3.91	0.464
Family history of atopy	4.99	1.99–12.53	0.001
Cassano Stage III–IV	1.13	0.47–2.71	0.788
High total IgE	1.12	0.39–3.27	0.831
Eosinophilia	1.12	0.41–3.05	0.822

(**A**) Results are presented as adjusted odds ratios (aORs), 95% confidence intervals (CIs), and *p*-values from a multivariable logistic regression model. Clinical atopy was defined as physician-diagnosed allergic rhinitis, asthma, atopic dermatitis, and/or food allergy. Cassano Stage III–IV indicates advanced adenoid hypertrophy [11]. Statistical significance was set at *p* < 0.05. (**B**) Results are presented as adjusted odds ratios (aORs), 95% confidence intervals (CIs), and *p*-values from a multivariable logistic regression model. IgE-mediated sensitization was defined as a positive skin prick test and/or allergen-specific IgE positivity. Cassano Stage III–IV indicates advanced adenoid hypertrophy [11]. Statistical significance was set at *p* < 0.05.

**Table 5:** Multivariable logistic regression analysis of factors associated with advanced adenoid hypertrophy in children.

Independent Variables	aOR	95% CI	*p* Value
Age (years)	0.97	0.89–1.06	0.550
Male sex	0.93	0.62–1.40	0.739
Passive smoking exposure	1.35	0.84–2.16	0.218
Family history of atopy	0.95	0.54–1.70	0.875
Eosinophilia	2.07	1.30–3.29	0.002
Elevated total IgE	0.96	0.53–1.75	0.894
Aeroallergen sensitization	1.08	0.61–1.93	0.787

Results are presented as adjusted odds ratios (aORs), 95% confidence intervals (CIs), and *p*-values. Advanced adenoid hypertrophy was defined as Cassano Stage III–IV. Associations are reported; causal inferences cannot be made due to the retrospective design.

## Data Availability

The original contributions presented in this study are included in the article/Supplementary Material. Further inquiries can be directed to the corresponding author.

## Supplementary Materials

The following supporting information can be downloaded at https://www.mdpi.com/article/10.3390/children13030374/s1. Table S1: Prevalence of atopic features and advanced adenoid hypertrophy by age group. Table S2: Multivariable logistic regression analyses of factors associated with advanced adenoid hypertrophy stratified by age group.
