# Supplementary material for: Atopic Features and Inflammatory Markers Across Cassano-Graded Adenoid Hypertrophy
Source: Children (Basel). 2026 Mar 6;13(3):374. doi: 10.3390/children13030374 (PMC13025132; doi:10.3390/children13030374)
Supplement: Supplementary file 1 [file children-13-00374-s001.zip › children-4179862-supplementary.pdf]

Table S1. Prevalence of atopic features and advanced adenoid hypertrophy by age group.

Age group (years)	n	Clinical atopy %	Sensitization %	Elevated IgE %	Eosinophilia %	Advanced AH %
3–5	148	24.3	18.2	15.5	20.9	34.5
6–8	172	26.7	22.1	17.4	26.7	44.2
9–12	106	35.8	31.1	16.0	38.7	37.7

Data are presented as percentages within each age group. Clinical atopy was defined as physician-diagnosed allergic rhinitis, asthma, atopic dermatitis, and/or food allergy. Sensitization refers to a positive skin prick test and/or allergen-specific IgE positivity. Elevated total immunoglobulin E (IgE) and eosinophilia were defined according to age-adjusted reference ranges. Advanced adenoid hypertrophy (AH) was defined as Cassano Stage III–IV.

Table S2. Multivariable logistic regression analyses of factors associated with advanced adenoid hypertrophy stratified by age group.

Variable	3–5 years aOR (95% CI)	P	6–8 years aOR (95% CI)	p	9–12 years aOR (95% CI)	p
Male sex	0.64 (0.31–1.31)	0.218	1.44 (0.76–2.76)	0.266	0.67 (0.28–1.59)	0.364
Passive smoking	1.43 (0.62–3.30)	0.401	1.20 (0.53–2.70)	0.664	1.48 (0.59–3.73)	0.401
Family history of atopy	0.68 (0.24–1.89)	0.456	1.12 (0.45–2.83)	0.805	1.02 (0.29–3.58)	0.977
Elevated total IgE	1.52 (0.54–4.30)	0.432	0.53 (0.20–1.43)	0.213	1.34 (0.38–4.66)	0.650
Aeroallergen sensitization	2.13 (0.74–6.12)	0.159	0.62 (0.24–1.59)	0.318	1.04 (0.33–3.32)	0.941
**Eosinophilia**	1.68 (0.70–4.02)	0.243	**3.54 (1.60–7.83)**	**0.002**	1.73 (0.70–4.29)	0.232

Values are presented as adjusted odds ratios (aORs) with 95% confidence intervals derived from multivariable logistic regression models within each age group. Advanced adenoid hypertrophy was defined as Cassano Stage III–IV. Models included sex, passive smoking exposure, family history of atopy, elevated total IgE, aeroallergen sensitization, and eosinophilia.
